# Editorial: RNA-Seq Analysis: Methods, Applications and Challenges

**DOI:** 10.3389/fgene.2020.00220

**Published:** 2020-03-17

**Authors:** Filippo Geraci, Indrajit Saha, Monica Bianchini

**Affiliations:** ^1^Institute for Informatics and Telematics, CNR, Pisa, Italy; ^2^Department of Computer Science and Engineering, National Institute of Technical Teachers' Training and Research, Kolkata, India; ^3^DIISM, University of Siena, Siena, Italy

**Keywords:** RNA-seq, algorithm, software pipeline, method assessment, differenial analysis

## 1. Introduction

RNA-seq has revolutionized the research community approach to studying gene expression. In fact, this technology has opened up the possibility of quantifying the expression level of all genes at once, allowing an ex post (rather than ex ante) selection of candidates that could be interesting for a certain study. The continuous drop in costs and the independence of library preparation protocols from the model species, have convinced the stakeholders to invest in this technology, by creating consortia able to produce large disease-specific datasets that, in turn, fostered transcriptomic research at a population level. Among many others, a virtuous example in this sense is The Cancer Genome Atlas. In a short time RNA-seq has moved from a technology to merely quantify the expression of genes to a powerful tool to: discover new transcripts (via *de novo* transcriptome assembly), characterize alternative splicing variants or new cell types (through single cell RNA sequencing). Leveraging on RNA-seq for daily diagnostic activities is no longer a dream but a consolidated reality.

Although established best practices exist, managing RNA-seq data is not easy. Before sequencing, it is essential to carefully plan library preparation in order to minimize downstream analysis biases. Budget optimization is another important factor. Sequencing multiple samples increases statistical power and reduces undesired side effects due to noise and variability. However, more samples imply higher costs. Multiplexing has proved to be an effective tool to limit the budget without sacrificing the number of samples. DNA barcoding enables combining up to 96 samples into a single line, trading a lower sequencing depth for a higher number of sequenced samples. The downside of this technique is the increased burden of data analysis to achieve the same accuracy that would be achieved with a richer input.

Downstream sequencing, fastq data must be validated and processed to distill raw reads into a quantitative measure of gene expression. While validation is somehow a standard procedure, read count depends on the type of RNA (microRNA, etc.) and on the target application. Usually reads are: subjected to adapter removal, aligned against a reference genome, grouped by functional unit (e.g., transcripts, genes, microRNA, etc.), normalized and counted. Subsequent analyses can vary dramatically according to the application. In the simplest setting, the subset of genes responsible for the differences on the phenotype between two populations should be discovered. In other cases, one may want to build the co-expression (or reverse expression) network in order to find interacting genes or a pathway related to a certain phenotype. Other applications involve the discovery of unknown cell types, the organization of cell types in homogeneous families, the identification of new molecules (e.g., new microRNA, long non-coding RNA, etc.), or the annotation of new variants or alternative splicing.

## 2. Research Topic Organization

This Research Topic is divided into three main sections: five articles cover the RNA-seq workflow, four papers discuss the most recent frontier of single cell RNA sequencing, while the last four contributions report on case studies, related to tumor profiling and plant science.

In the first part, we attempted to analyze the RNA-seq process (from experimental design to analysis and extraction of new knowledge) by highlighting the key choices of the state-of-the-art workflows. Although we have mainly focused on computational aspects, we believe that this Research Topic can catch the interest of those readers, specialized in the field of life science, who intend to become independent and autonomous in the analysis of their own data. Two papers of this section describe new methods: for the identification of differentially expressed genes and for the prediction of the circRNA coding ability.

The second section introduces a recent branch of RNA-seq data analysis: single cell sequencing (scRNA-seq). Although conceptually similar to sequencing cells in bulk, the single cell resolution of this technique introduces a lot of noise, that requires *ad hoc* analysis methods. Much of this section is dedicated to the introduction of basic single cell RNA sequencing concepts, from laboratory protocols to the most common analyses. In particular, the problems of assessing the results of clustering cell types and the reproducibility of differential expression experiments are discussed. Finally, this section concludes with the description of a new method to infer missing counts due to poor coverage of sequencing.

The last part of the Research Topic was dedicated to four case studies: three concerning tumors and one application in plant science. The rationale behind this choice was that of showing different types of analysis. In the conceptually simpler case, the goal of the analysis was to create a panel of genes prognostic of the onset of cancer. Next, an example of a co-expression network is shown. Finally, an example of interaction among different types of RNA (long non-coding, genes, microRNAs) has been reported, showing the complexity of the pathways that regulate the life of cells.

### 2.1. RNA-Seq Analysis

In Reed et al., the opportunity offered by Multiplexed RNA Sequencing is discussed. The study provides a comparison of several methods using real data from immortalized human lung epithelial cells.

In Peri et al., RMTA, an user-friendly analysis workflow, is proposed. RMTA was designed to provide standard pre-processing tools (i.e., read quality analysis, filters for lowly expressed transcripts, and read counting for differential expression analysis) in a scalable and easy to deploy environment.

In Jimenez-Jacinto et al., an integrative differential expression analysis web server (IDEAMEX) is described. The rationale of IDEAMEX is that of freeing non-expert users from the (sometimes frustrating) experience of interacting with the UNIX-based environment for standard differential expression analyses.

In Gao et al., a new method for the identification of differentially expressed genes is reported. The key observation of this work is that the binomial distribution at the basis of the majority of the algorithms for differential expression analysis is unable to capture underdispersion characteristics of RNA-seq data.

In Sun and Li, the problem of predicting whether a given circular RNA can be translated or not is investigated. Circular RNAs differ from other types of RNA in that they are arranged as rings joining 3′ and 5′ endpoints. This characteristic makes hard to decide about their translation potential. The manuscript provides an algorithm to identify the coding ability of circRNAs with high sensitivity.

### 2.2. Single Cell RNA Sequencing

In Chen et al., an overview of currently available single-cell isolation protocols and scRNA-seq technologies is provided. In addition, several methods for scRNA-seq data analysis, from quality control to network reconstruction, are discussed.

In Krzak et al., the use of clustering to study heterogeneity of cells is dissected. In particular, this work aims at providing new insights into the advantages and drawbacks of scRNAseq clustering, highlighting open challenges.

In Mou et al., some issues connected to the reproducibility of differential expression studies is debated. The complexity of this type of analyses stands in the paucity of RNAs and in the consequent lower signal to noise ratio. The article shows pros and cons of standard and *ad-hoc* software for differential expression.

In Mongia et al., a method to impute dropouts in single cell expression data is detailed. Experiments on real data show that the proposed software is able to discriminate the real absence of reads from dropout events.

### 2.3. Case Studies

In Yin et al., differential expression analysis is used to pinpoint a small panel of genes potentially prognostic for the onset of Glioblastoma. The focus of the article is that of improving healthy/diseased classification regardless of the interaction among genes.

In Zhu et al., co-expressed genes are identified in order to build a network of interactions. Subsequently, the network is analyzed to select hub genes associated with soft tissue sarcomas.

In Zheng et al., the dynamics of the interaction among different molecules in lung adenocarcinoma is studied. The article reports on how the dysregulation of a long non-coding RNA triggers a sequence of dysregulations, causing the cell cycle arrest.

In Tengkun et al., genomics and trascriptomics data are integrated in order to identify the crucial genes that affect anthocyanin biosynthesis transforming quantitative traits into quality traits.

## Author Contributions

The authors all contributed equally to the Research Topic assembly and editing and to this editorial.

### Conflict of Interest

The authors declare that the research was conducted in the absence of any commercial or financial relationships that could be construed as a potential conflict of interest.

